# Surgery

**Published:** 1899-03

**Authors:** J. Lacy Firth


### SURGERY.

**Thrombosis and embolism of the mesenteric vessels,** as a cause of acute abdominal affections, has hardly received the amount of general attention it deserves, and it may be worth while, therefore, to draw attention to the signs and symptoms it produces.

The usual result of occlusion of the mesenteric vessels is infarction of the intestine. This infarction may follow occlusion of either the arteries or veins, the effect generally being greater when the latter are obstructed, for in that event there is no hindrance to the supply of blood by the artery but there is hindrance to the escape, and of necessity there is produced intense vascular engorgement and often extravasation of blood in the affected area. When the superior or inferior mesenteric artery is blocked, engorgement also follows, the flow of blood producing it in this case being from the anastomosing arteries and from the veins, the backward pressure of the latter coming into play when the *vis a tergo* is cut off. Litten's experimental ligation of the superior mesenteric artery near its origin, in dogs, always produced this result.

Though infarction of the intestine and mesentery, with gangrene in the severest, and ulceration or simple congestion in the mildest, cases, most frequently follows occlusion of the superior mesenteric artery: yet in 2 cases observed by Virchow, and 1 by Tiedemann, that artery was found obliterated without injury to the intestine.[2] Chiene[3] has also reported a case met with in the dissecting-room, in which there was occlusion of the three anterior branches of the abdominal aorta supplying the viscera.

[1] *Lancet*, 1899, i. 286. [2] Quoted by Elliot, *Ann. Surg.*, 1895, xxi. 9.

[3] *J. Anat. & Physiol.*, 1869, iii. 65.

The vitality of the intestine had in no way been impaired, having been maintained by the dilatation of collateral blood-vessels. It appears from these cases that the intestine escapes injury from interference with normal channels of blood-supply if the interference is sufficiently gradually produced to allow of compensatory dilatation of anastomosing arteries.

Councilman[1] remarks upon the probability that small emboli frequently enter the superior mesenteric artery, their entry being favoured by the size of that artery and the angle it forms with the aorta; but that the free anastomosis between its small branches prevents these small emboli impairing intestinal nutrition. He reports 3 cases of embolism of the superior artery which he had met with. One of these is particularly interesting, for clinically the symptoms were those of complete intestinal obstruction, with vomiting finally becoming stercoraceous, great abdominal pain, and tympanites. The patient, who was 85 years of age, died on the twelfth day of illness. The intestine presented a few small ecchymoses, but there was neither intense congestion nor at any place complete infarction. A thrombus attached to an atheromatous patch on the anterior wall of the aorta, just above the origin of the superior mesenteric artery, almost completely occluded the opening of that branch. He thinks it probable that the blood-supply through the obstructed vessel, though sufficient to maintain the vitality of the intestine, interfered with its innervation, leading to paralysis of the bowel and the attendant intestinal obstruction. In a second case, though the entire superior mesenteric artery was obstructed, there was only a beginning of infarction in a small area of the intestine.

Dr. Elliot[2] has analysed the records of 20 carefully reported cases of **occlusion of the superior mesenteric artery,** in which the **abdominal symptoms** were not overshadowed by those of heart disease or by coma. All the cases were rapidly fatal. Pain, often colicky, was the most prominent symptom, and occurred in nearly all. A very notable symptom was the presence of blood in the stools. This was recorded in 13 cases. In 3 the blood was passed without fæces. In 6 the hemorrhage was profuse. In 3 other cases neither fæces nor flatus were passed. Hæmatemesis occurred in 3. Blood is usually found in the intestines at the autopsy, even if not passed during life, and is also frequently found in the peritoneal cavity. In 2 cases there was a palpable swelling, which proved to be infarcted bowel; and in another, a swelling was due to a collection of blood in the mesentery. Subnormal temperature and extreme pallor were noticed in several cases, due no doubt to hemorrhage and peritoneal shock. In nearly half the cases there was diarrhœa. In some there was neither diarrhœa nor other disturbance of the bowels.[3] The source of the embolism is usually

[1] *Boston M. & S. J.*, 1894, cxxx. 410. [2] *Loc. cit.*
[3] F. S. Watson, *Boston M. & S. J.*, 1894, cxxxi. 555.

vegetations on the cardiac valves, or on atheromatous patches in the aorta. Watson, analysing 8 cases of total occlusion of the main trunk of the superior mesenteric artery, finds that in 1 case there was entire absence of abdominal symptoms, and in 2 others they were very slight, though the infarction affected a large part of the intestines. On the other hand, in a case in which only a small portion of the intestine was in a state of infarct, abdominal pain was sudden and violent, and there was profuse bloody diarrhœa. It is obvious, therefore, that from the clinical signs it is impossible to tell the extent or degree of the intestinal lesion.

It will have been noticed that one of the most characteristic symptoms mentioned above was bloody diarrhœa. If there is evidence of embolism in other parts of the body, producing for example gangrene of the feet, or hemiplegia, hæmoptysis and chest pain, or splenic or renal trouble, the diagnosis of the condition we are discussing will be much facilitated; but this evidence is often wanting. Again, the presence of heart disease and atheromatous arteries will help in the diagnosis. Watson thinks it possible to diagnose about one-fourth of the cases. The affection, perhaps, most closely resembles intussusception, as pointed out by Elliot.[1] In both infarction of the intestine and intussusception there is often diarrhœa and intestinal hemorrhage, and pain and vomiting. The hemorrhage is apt to be more profuse in the embolic affection, but has been recorded as the principal cause of death in both. In intussusception tympanites is rare in the early stages. In that affection too the patients are usually younger. According to Fitz, 56 per cent. of the cases of intussusception are under the age of 10. The characters of a tumour if present would be an important guide to the diagnosis. A few cases are recorded of embolism of the inferior mesenteric artery, but the symptoms do not sufficiently differ from those in which the superior artery is affected to permit of clinical differentiation. It has been said that the presence of fresh blood in the dejections, and the localisation of the pain in the lower part of the abdomen, point to embolism of the inferior vessel.[2]

Elliot[3] has found 14 reported cases of **thrombosis of the superior mesenteric vein.** They were even more rapidly fatal than those of embolism of the artery, nearly all having died on the second or third day with symptoms of intestinal obstruction. The symptoms of this affection are much the same as those of embolism of the corresponding artery, with the exception that diarrhœa has not been recorded as present in any. Blood was vomited or passed in large quantity by the bowel in half the cases. In 5 of the cases there was cirrhosis of the liver, and 2 were syphilitic. In 2 cases a volvulus was considered to be the result of the thrombosis.

It may, therefore, be said that acute abdominal symptoms,

[1] *Loc. cit.* [2] Elliot, *Loc. cit.* [3] *Loc. cit.*

associated with the passage of blood and with portal obstruction from cirrhosis of the liver or other cause, would suggest the diagnosis of thrombosis of the mesenteric veins.

The **prognosis** in these affections of the mesenteric vessels is very bad, though, as mentioned above, there are records which show that patients have survived.

Elliot has only found in literature 3 cases which have been operated upon. He reports 2 additional cases of his own. In none was the diagnosis previously made; and 4 out of the 5 died unrelieved.

Laparotomy, and resection of the intestine if it be found to to require it, is the only **treatment** that offers any prospect of success. Watson states that the records of a number of autopsies showed that one-sixth of the cases might have been relieved by such treatment. In the single successful operation for this condition on record, that of Elliot,[1] four feet of intestine, almost gangrenous from infarction, were removed. The open ends of the intestine were sutured to the abdominal wound, and the artificial anus thus produced was subsequently closed by a second operation. The patient was a male, aged 25, and the cause of the infarction was thrombosis of the mesenteric veins. Elliot's second case was one of thrombosis of a branch of the mesenteric artery. An artificial anus was made in the descending colon; but the patient died. At the autopsy four inches of the intestine were found gangrenous and to have been perforated. In most cases the presence of heart disease, atheroma, or cirrhosis of the liver, militates strongly against surgical success.

In all cases of **rupture of the kidney**, whether open or subcutaneous, when first seen the question of the advisability of exploring or of performing a primary nephrectomy will arise. Morris[2] and Keen[3] have each written papers, in which this matter is helpfully considered. They are agreed that the symptom of hæmaturia rarely affords help in deciding the question, for it is a very unreliable guide as to the severity of the renal injury. Hæmaturia may be considerable in degree when the renal injury is slight, and slight or absent when the injury is most severe. If, however, hæmaturia is exhausting the patient by its severity, and especially if bloodclots with their attendant septic dangers accumulate in the bladder, then and then only does this symptom afford clear indications that the kidney should be explored. The clots in the bladder must be removed, either by expression (Willard) if laparotomy is performed, otherwise by the aid of a catheter or evacuator, and their further formation must be prevented, either by ligature of the bleeding-points in the kidney or plugging the wound in it, or by nephrectomy. Again, primary anuria does not afford an indication for operation unless it persists for twenty-four or thirty-six hours after shock has passed off, or unless it appears after the lapse of

[1] *Loc. cit.* [2] *Clin. J.*, 1894, iv. 217. [3] *Ann. Surg.*, 1896, xxiv. 138.

some days. In these exceptional circumstances anuria indicates the advisability of exploration, for it may be due to a ureteral clot preventing the escape of the secretion of the only functionally active kidney present, the other kidney being either absent or diseased. Life in such cases can only be saved by providing an escape for the urine through the loin.

By what **symptoms** or signs then are we to be guided in the majority of cases, in deciding whether to operate or not in these cases of subcutaneous rupture of the kidney? It is "the presence or absence of a distinct and increasing swelling in the loin" which affords the surest guide. As Morris puts it:—"The presence of a distinct and increasing swelling in the renal region makes an early incision through the loin imperative: it is the only way to cut short the progress of the case, to limit the extravasation into the peri-renal tissues, to check or prevent suppuration, to prevent sloughing of the peritoneum, and to save the life of the patient." Having exposed the kidney, it will have to be either removed at once, or dealt with as described above, the degree of injury affording the correct indications.

If in the first instance palliative and non-operative treatment has been adopted, and the case does not progress favourably, and it is obvious that a large hæmatoma has gradually formed, then a secondary exploratory operation, with or without secondary nephrectomy, must be performed. Secondary nephrectomy becomes much more dangerous if postponed until septic complications have arisen. The exploratory lumbar incision should be made to anticipate or prevent septic complications if possible. A good instance of a successful secondary nephrectomy performed on the above principles is one published by Moynihan,[1] in which the operation was done on the twelfth day after an injury which had torn the kidney from the cortex to the pelvis, almost completely dividing it into two parts. There were no septic complications. Page[2] reports another case of secondary nephrectomy which, though ultimately successful, illustrates the disadvantage of postponing these operations until septic complications have arisen. The patient passed through a long and critical illness, and some months after convalescence there was still slight pyuria, suggesting the possibility of infective processes continuing in the other kidney.

J. LACY FIRTH.

### OPHTHALMOLOGY.

The use of a small electro-magnet in extracting **foreign bodies** which have penetrated the eyeball has been resorted to for more than twenty years. In 1892 Haab, acting upon the principle that "He that has the strongest magnet will obtain the best results," invented a larger and immensely more powerful instru-

[1] *Quarterly M. J.*, 1897–98, vi. 246. [2] *Clin. J.*, 1896–97, ix. 65.

